# Transcriptomic and metabolomic analysis provides insights into anthocyanin and procyanidin accumulation in pear

**DOI:** 10.1186/s12870-020-02344-0

**Published:** 2020-03-27

**Authors:** Zhen Zhang, Changping Tian, Ya Zhang, Chenzhiyu Li, Xi Li, Qiang Yu, Shuo Wang, Xinyu Wang, Xuesen Chen, Shouqian Feng

**Affiliations:** 1 State Key Laboratory of Crop Biology, Shandong Agricultural University, No.61, Daizong Road, Tai’an, 271018 China; 2 College of Horticulture Sciences, Shandong Agricultural University, No.61, Daizong Road, Tai’an, 271018 China; 3 Cherry Research Department, Yantai Agricultural Science and Technology Institute, No.26, West Gangcheng Street, Yan’tai, 265500 China

**Keywords:** Pear, Anthocyanin, Procyanidin, Metabolome and transcriptome analyses, *PcGSTF12*, *PcMYB114*

## Abstract

**Background:**

Pear is one of the most important fruit crops worldwide. Anthocyanins and procyanidins (PAs) are important secondary metabolites that affect the appearance and nutritive quality of pear. However, few studies have focused on the molecular mechanism underlying anthocyanin and PA accumulation in pear.

**Results:**

We conducted metabolome and transcriptome analyses to identify candidate genes involved in anthocyanin and PA accumulation in young fruits of the pear cultivar ‘Clapp Favorite’ (CF) and its red mutation cultivar ‘Red Clapp Favorite’ (RCF). Gene–metabolite correlation analyses revealed a ‘core set’ of 20 genes that were strongly correlated with 10 anthocyanin and seven PA metabolites. Of these, *PcGSTF12* was confirmed to be involved in anthocyanin and PA accumulation by complementation of the *tt19–7 Arabidopsis* mutant. Interestingly, *PcGSTF12* was found to be responsible for the accumulation of procyanidin A3, but not petunidin 3, 5-diglucoside, opposite to the function of AtGSTs in *Arabidopsis*. Transformation with *PcGSTF12* greatly promoted or repressed genes involved in anthocyanin and PA biosynthesis, regulation, and transport. Electrophoretic mobility shift and luciferase reporter assays confirmed positive regulation of *PcGSTF12* by PcMYB114.

**Conclusion:**

These findings identify a core set of genes for anthocyanin and PA accumulation in pear. Of these, *PcGSTF12*, was confirmed to be involved in anthocyanin and PA accumulation. Our results also identified an important anthocyanin and PA regulation node comprising two core genes, *PcGSTF12* and *PcMYB114*. These results provide novel insights into anthocyanin and PA accumulation in pear and represent a valuable data set to guide future functional studies and pear breeding.

## Background

Pear is an important fruit for human consumption, and its total global production is ranked third after grape and apple [1]. Pear is cultivated commercially in 76 countries or regions worldwide [2], among which China is the world’s leading pear producer. In 2017, China produced 16.4 million tons (Mt) of pear fruits, accounting for 68% of global pear production (24.2 Mt) (FAOSTAT, 2017).

Pears are a good source of anthocyanin and procyanidin (PA) metabolites. To date, five anthocyanins (cyanidin 3-galactoside, cyanidin 3-glucoside, cyanidin 3-arabinoside, peonidin 3-galactoside, and peonidin 3-glucoside) and two PAs (procyanidin B1 and procyanidin B2) have been identified in the red pear cultivars ‘D’Anjou’ [3] and ‘Red Zaosu’ [4]. Anthocyanins and PAs are abundant in the skin of pear fruits, they contribute to their color, taste, and nutrition [5].

The anthocyanin and PA biosynthesis pathways have been well characterized in plants. Anthocyanin and PA are initially biosynthesized from phenylalanine, and share most steps in the flavonoid biosynthetic pathway. They are biosynthesized in the cytosol by enzymes including phenylalanine ammonia lyase (PAL), chalcone isomerase (CHI), chalcone synthase (CHS), flavonoid 3-hydroxylase (F3H), and dihydroflavonol reductase (DFR) [6]. Leucoanthocyanidins and anthocyanidins are two important branch points between the anthocyanin and PA biosynthesis pathways. Downstream of these branch points, anthocyanins are synthesized by anthocyanidin synthase (ANS) and UDP-glucose flavonoid 3-Oglucosyl transferase (UFGT), and PAs are synthesized by leucoanthocyanidin reductase (LAR) and anthocyanidin reductase (ANR) [7, 8]. O-methyltransferase (OMT) and glycosyltransferase (GT) are responsible for the elaboration of diverse anthocyanins and PAs [9, 10]. After anthocyanins and PAs are synthesized in the cytosol, they are transported to their final destination, the vacuole. Some glutathione S-transferases (GSTs) and multidrug and toxic compound extrusion proteins (MATEs) are believed to function as anthocyanin and PA carrier proteins to sequester them into vacuoles [11].

The molecular mechanism underlying anthocyanin and PA accumulation has been extensively studied in numerous plants. Many anthocyanin and PA structural genes and their upstream regulators have been identified and characterized. Of these, R2R3-MYB TFs play key roles in controlling anthocyanin and PA accumulation by acting together with bHLH and WD40 proteins to regulate structural genes. Genes encoding R2R3-MYB TFs that contribute to anthocyanin accumulation include *MdMYB1* in apple, *PyMYB10* and *PyMYB114* in pear, and *VvMYBA1* in grape [12–15]. Some R2R3-MYB TFs regulate both anthocyanins and PAs, including *VvMYB5a* and *VvMYB5b* in grape, *MdMYB9* and *MdMYB11* in apple, *PbMYB10b* and *PbMYB9* in pear, and *PpMYB18* in peach [4, 16–19]. Other R2R3-MYB TFs are related only to the regulation of PAs, including *VvMYBPA1* in grape and *PpMYBPA1* in peach [16, 20]. In addition, other TFs such as AUX and ERF also regulate anthocyanin or PA biosynthesis by directly or indirectly interacting with R2R3-MYB TFs and structural genes [21, 22].

Recent technical advancements in transcriptome and metabolome analyses have provided effective ways to identify new genes and metabolites, and to elucidate complex secondary metabolic bioprocesses in plants. In fig (*Ficus carica* L.), integrated transcriptome and metabolome analyses have revealed genes in flavonoid and anthocyanin pathways that show differential expression between purple- and green-skinned cultivars [23]. Another study using combined transcriptome and metabolome datasets successfully constructed expression–anthocyanin metabolite networks in potato [24]. Recently, seven flavonoid metabolites and six genes were identified as candidates associated with the pigmentation of the red-fleshed and green-fleshed cultivars of *Actinidia arguta* [25].

Red pears have high nutritional and economic value because they are rich in anthocyanins and PAs. Therefore, studies on the regulation of anthocyanins and PAs are of great interest for the improvement of anthocyanin and PA production in pears. Considering the large number of anthocyanins and PAs, the molecular mechanisms of their biosynthesis and embellishment in pear might be more complex than expected. Bud mutation is an important method of selecting new red pear varieties. Red mutations are ideal materials to study the molecular mechanism of anthocyanin and PA accumulation because of their highly similar genetic backgrounds [13]. In the present study, we carried out metabolome and transcriptome analyses to identify candidate genes involved in anthocyanin and PA accumulation in pear using the young fruits of ‘CF’ and its red mutant ‘RCF’. Correlation analyses between differentially expressed genes (DEGs) and anthocyanins/PAs revealed 203 candidate genes for the accumulation of 10 anthocyanins and seven PAs in pear. Of these, 20 genes were strongly correlated with all 10 anthocyanins and seven PAs. Thus, they seemed to be the core candidate genes related to anthocyanin and PA accumulation in pear. The *GST* gene *PcGSTF12* was correlated with most anthocyanin and PA metabolites. *PcGSTF12* was confirmed to play an important role in anthocyanin and PA accumulation in pear by functional complementation analyses. In addition, *PcGSTF12* was found to be directly and positively regulated by PcMYB114, a well-known TF regulating anthocyanin accumulation in pear. These results greatly extend our knowledge of the molecular mechanism of anthocyanin/PA accumulation in pear.

## Results

### Anthocyanin and PA profiles of ‘CF’ and its red mutant ‘RCF’

**Fig. 1:**
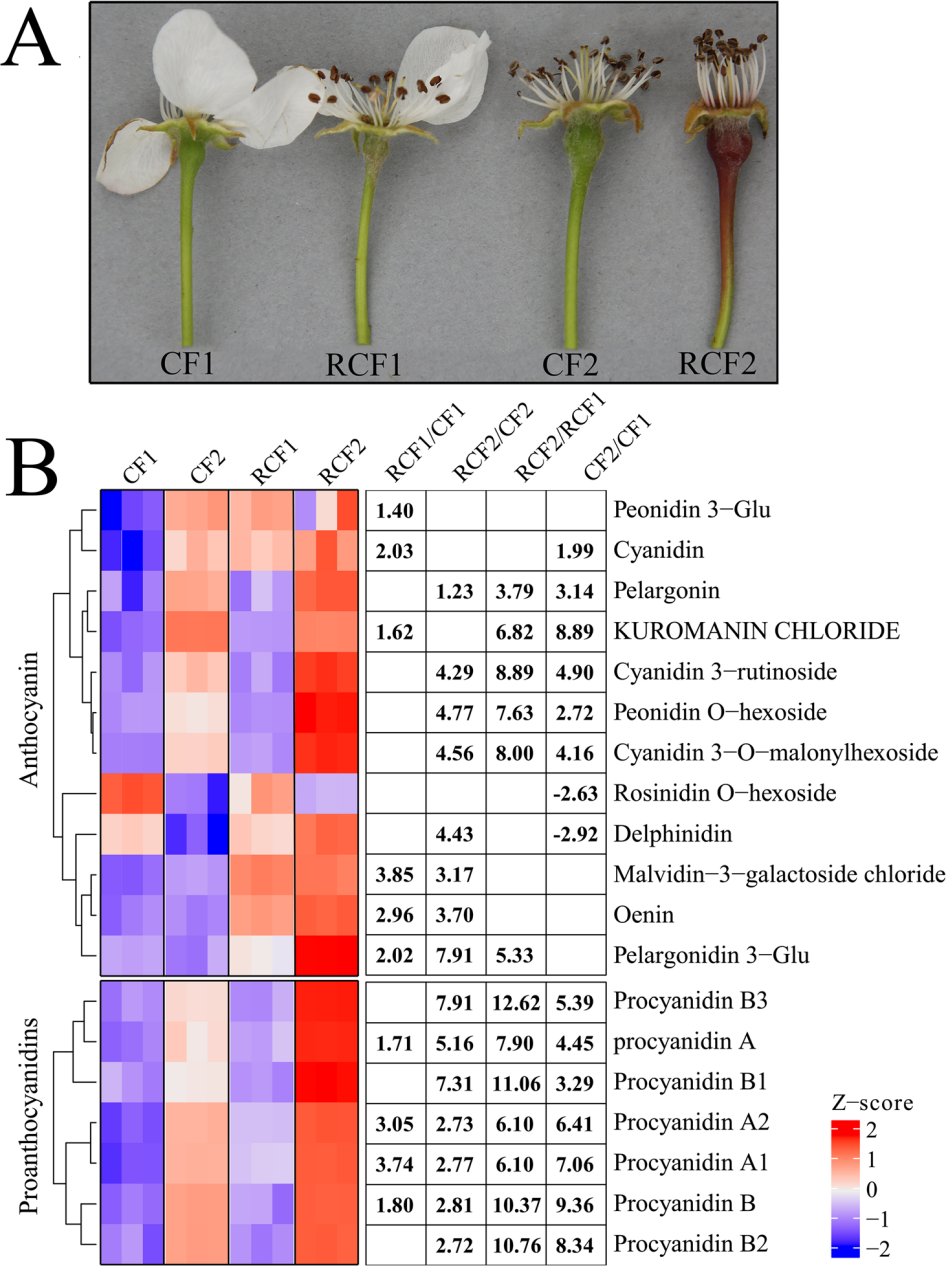
Changes in pigment (anthocyanins and procyanidins; PAs) contents in ‘CF’ and ‘RCF’. **a** Changes in fruit pigmentation of ‘CF’ and ‘RCF’. **b** Concentrations of anthocyanins and PAs in peel of ‘CF’ and ‘RCF’. ‘CF1’ and ‘RCF1’ refer to fruits of ‘CF’ and ‘RCF’ collected at 2 days after full bloom (DAFB), respectively, ‘CF2’ and ‘RCF2’ refer to fruits of ‘CF’ and ‘RCF’ collected at 5 DAFB, respectively. Numbers refer to -fold change in metabolite contents

Except for skin color, no significant morphological differences were observed between the fruits of ‘CF’ and its red mutant ‘RCF’. The young fruits of both ‘CF’ and ‘RCF’ initially had a deep green appearance. The color difference between ‘CF’ and ‘RCF’ became visible from about 5 days after full bloom (DAFB). The ‘RCF’ fruits quickly turned dark red, and retained their strong color until maturity. In contrast, the fruits of ‘CF’ only developed a slight red blush on the sun-exposed surface (Fig. 1a).

We identified and quantified the individual anthocyanins and PAs in ‘CF’ and its red mutant ‘RCF’. We identified and quantified 12 anthocyanins and seven PAs from ‘CF1’, ‘RCF1’, ‘CF2’, and ‘RCF2’. The anthocyanin metabolites included pelargonin, cyanidin 3-rutinoside, pelargonidin 3-Glu, malvidin-3-galactoside chloride, cyanidin 3-O-malonylhexoside, oenin, delphinidin, peonidin O-hexoside, cyanidin, and rosinidin O-hexoside; and the PAs included procyanidin A, procyanidin B3, procyanidin B, procyanidin A1, and procyanidin A2, which were all detected for the first time in pear. As shown in Fig. 1b, most anthocyanins and PAs were significantly up-regulated in ‘RCF’ compared with ‘CF’. The levels of anthocyanins and PAs in ‘CF’ and ‘RCF’ were initially low and then sharply increased during fruit coloration, except for delphinidin and rosinidin O-hexoside (Fig. 1b). These patterns of pigment accumulation were consistent with the strikingly different fruit color phenotypes of ‘RCF’ and ‘CF’.

The thresholds for significant differences in metabolite levels between the two cultivars were variable importance in projection (VIP) value ≥1 and | log2(fold change) | ≥ 1. Against these criteria, six, eight, six, and eight anthocyanin metabolites, and four, seven, seven, and seven PA metabolites were significantly differentially accumulated in the four comparison groups: ‘RCF1’ vs. ‘CF1’, ‘RCF2’ vs. ‘CF2’, ‘RCF2’ vs. ‘RCF1’, and ‘CF2’ vs. ‘CF1’, respectively (Supplementary Table S1). Therefore, these anthocyanins and PAs were selected for further metabolite and transcript correlation analyses.

### Transcriptome analysis

The RNA-seq process yielded 95.6 G clean bases and 637 million clean reads. The mean number of clean reads per sample was 53 million. Of the clean reads, 93.54% were mapped in total, and 90.72% were mapped uniquely against the improved apple reference genome sequence. In total, 14,514 genes were expressed with FPKM ≥10 (Supplementary Table S2).

**Fig. 2:**
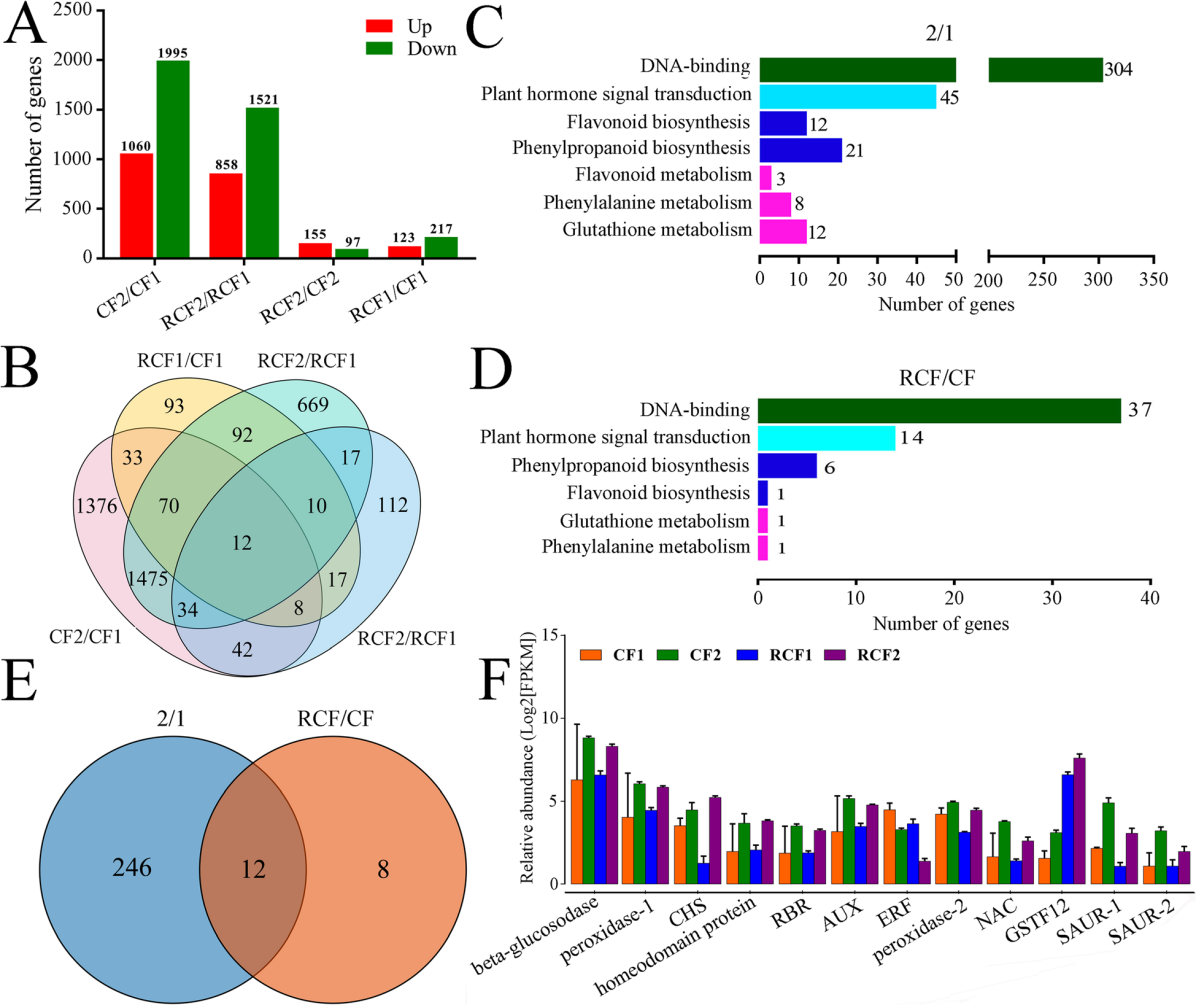
Number of differentially expressed genes (DEGs) identified by RNA-seq analysis. **a** Numbers of DEGs. **b** Venn diagram representing numbers of DEGs. **c** Number of DEGs in group 2–1 categorized into DNA binding, plant hormone signal transduction, flavonoid biosynthesis, phenylpropanoid biosynthesis, brassinosteroid biosynthesis, zeatin biosynthesis, flavonoid metabolism, phenylalanine metabolism, glutathione metabolism and ABC transporters. **d** Number of DEGs in group RCF-CF involved in DNA binding, plant hormone signal transduction, phenylpropanoid biosynthesis, flavonoid biosynthesis, glutathione metabolism and phenylalanine metabolism. **e** Overlapping DEGs between group 2–1 and group RCF-CF. **f** Transcript levels of overlapping DEGs between group 2–1 and RCF-CF as determined by RNA-seq

We identified 4065 DEGs in the four comparison groups. There were 340 DEGs in ‘RCF1’ vs. ‘CF1’, 252 in ‘RCF2’ vs. ‘CF2’, 2379 in ‘RCF2’ vs. ‘RCF1’, and 3055 in ‘CF2’ vs. ‘CF1’ (Supplementary Table S3): in those comparison groups, 123, 155, 858, and 1060 genes were up-regulated, and 217, 97, 1521, and 1995 genes were down-regulated, respectively (Fig. 2a). Only 12 DEGs were common to all four comparison groups (Fig. 2b).

The DEGs between group 2–1 and group RCF-CF were subjected to GO (Supplementary Table S4) and KEGG functional pathway analyses (Supplementary Table S5). For correlation tests with anthocyanins and PAs, we chose DEGs in group 2–1 categorized into DNA binding, plant hormone signal transduction, flavonoid biosynthesis, phenylpropanoid biosynthesis, flavonoid metabolism, phenylalanine metabolism, glutathione metabolism, and DEGs in group RCF-CF categorized into DNA binding, plant hormone signal transduction, phenylpropanoid biosynthesis, flavonoid biosynthesis, glutathione metabolism, and phenylalanine metabolism (Fig. 2c, d). In total, we selected 203 DEGs. Of these, there were 12 DEGs that overlapped between group 2–1 (‘RCF2’ vs. ‘RCF1’, and ‘CF2’ vs. ‘CF1’) and group RCF-CF (‘RCF1’ vs. ‘CF1’, ‘RCF2’ vs. ‘CF2’). These 12 DEGs encoded a β-glucosidase (PCP011059), two peroxidases (PCP024451 and PCP017906), a CHS (PCP023048), a homeodomain protein (PCP024513), an ERF (PCP044584), a RBR (retinoblastoma-related protein, PCP007207), an AUX (PCP036703), a NAC (PCP028501), a GST (PCP025171), and two SAURs (PCP037299 and PCP040169) (Fig. 2e). Of these, *PcGST* (PCP025171) was the most up-regulated gene in the comparison groups ‘RCF1 *vs*. CF1’ and ‘RCF2 *vs*. CF2’ (Fig. 2f).

### Correlation analysis between selected transcripts and anthocyanins/PAs

To identify the candidate genes in anthocyanin and PA accumulation in pear, we conducted correlation analyses between selected transcripts and metabolites. In total, we detected 420 significant correlations (correlation coefficient, R^2^ > 0.8) between 203 transcripts and 17 metabolites, including 10 anthocyanins (kuromanin chloride, pelargonin, cyanidin 3-rutinoside, pelargonidin 3-Glu, malvidin-3-galactoside chloride, cyanidin 3-O-malonylhexoside, oenin, delphinidin, peonidin O-hexoside, cyanidin, and rosinidin O-hexoside) and seven PAs (procyanidin A, procyanidin A1, procyanidin A2, procyanidin B, procyanidin B1, procyanidin B2, and procyanidin B3) (Supplementary Table S6). Each metabolite was correlated with many different transcripts. Malvidin-3-galactoside chloride, oenin, and delphinidin were correlated with the fewest transcripts: five, seven, and three transcripts, respectively. Kuromanin chloride and pelargonin were correlated with the highest numbers of transcripts: 184 and 56 transcripts, respectively. Interestingly, kuromanin chloride and pelargonin shared the largest number of common transcripts (36 transcripts). This suggested that kuromanin chloride and pelargonin might have evolved similar accumulation mechanisms.

**Fig. 3:**
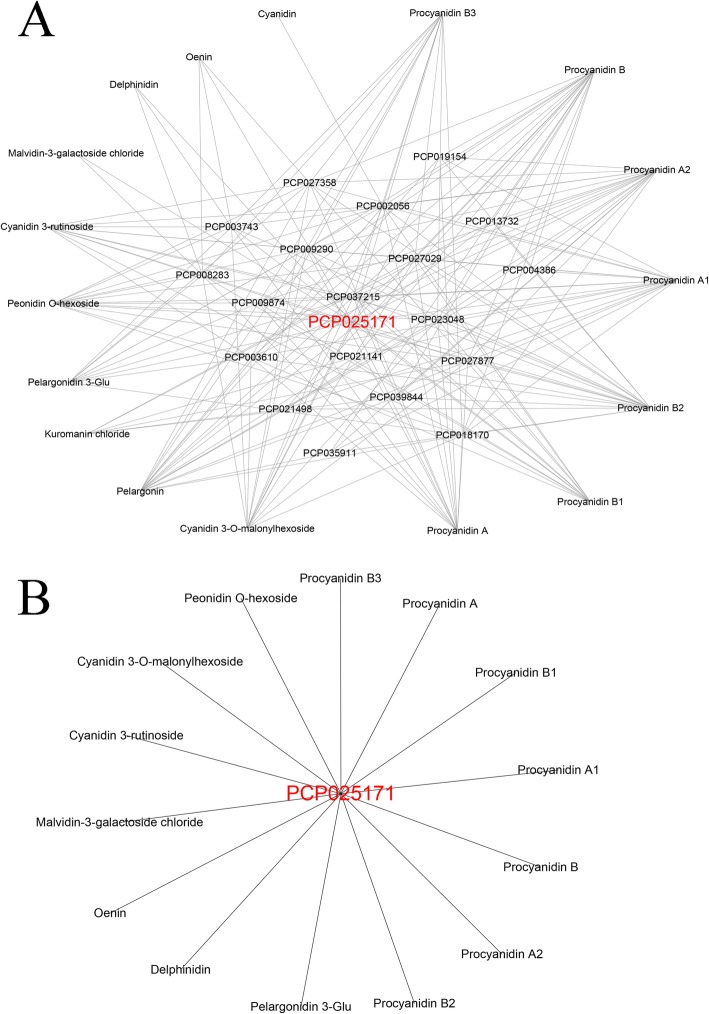
Connection network between core genes and anthocyanin and procyanidin (PA) metabolites. **a** Networks between 20 core genes and 10 anthocyanins and seven PAs. **b** Networks between *PcGSTF12* (PCP025171) and seven anthocyanins and seven PAs. *PcGSTF12* is shown in red font

The 203 transcripts were annotated with descriptions from the SwissProt and NR databases. Six transcripts have been functionally characterized to play roles in anthocyanin accumulation in pear previously: *PcMYB10*, *PcMYB114*, *PcCHS*, *PcCHI*, *PcF3H,* and *PcANS* (Supplementary Table S7). The rest were newly identified as candidate genes involved in anthocyanin and PA accumulation in pear. The 203 transcripts were grouped into two clusters (I-II) (Supplementary Table S7). Genes in cluster I were strongly correlated with anthocyanins. Cluster I comprised 183 genes (90.1%). Of these, 147 genes were correlated with a single anthocyanin: 142 genes were correlated with kuromanin chloride, three genes were correlated with cyanidin, one gene was correlated with malvidin-3-galactoside chloride, and one gene was correlated with pelargonin. The remaining genes in cluster I were closely correlated with two or more anthocyanins: 31 genes were commonly correlated with kuromanin chloride and pelargonin, two genes were correlated with pelargonin and cyanidin, two genes were correlated with malvidin-3-galactoside chloride and oenin, and one gene was correlated with cyanidin 3-rutinoside, oenin, and cyanidin. Cluster II contained 20 genes (9.9%) that were strongly correlated with both anthocyanins and PAs. Of these genes, two phenylpropanoid structural genes (encoding 4CL1 and 4CL2), six flavonoid structural genes (encoding CHS, 3 CHIs, F3H, and ANS), six TF genes (encoding bZIP1, MYB3, MYB86, MYB111, MYB114, and KNAT1), two phytohormone signal transduction genes (encoding IAA13 and ERF003), two DNA-directed RNA polymerase genes (encoding rpoB and Rpb1) and one GST transporter gene (encoding GSTF12) were positively correlated with anthocyanins and PAs. One WRKY TF gene, *WRKY28,* was negatively correlated with anthocyanins and PAs (Supplementary Table S7, S8). Each gene in cluster II was strongly correlated with many metabolites. We found that these 20 genes were strongly correlated with all 17 anthocyanin and PA metabolites (Fig. 3a). Thus, they were considered to represent the core genes for anthocyanin and PA accumulation in pear. Of these, *PcRPB1* (PCP004386) was correlated with the fewest metabolites: one anthocyanin and three PAs; and *PcGSTF12* (PCP025171) was correlated with the most metabolites: seven anthocyanins and seven PAs (Fig. 3b).

### qPCR analysis of DEGs related to anthocyanin and PA accumulation

**Fig. 4:**
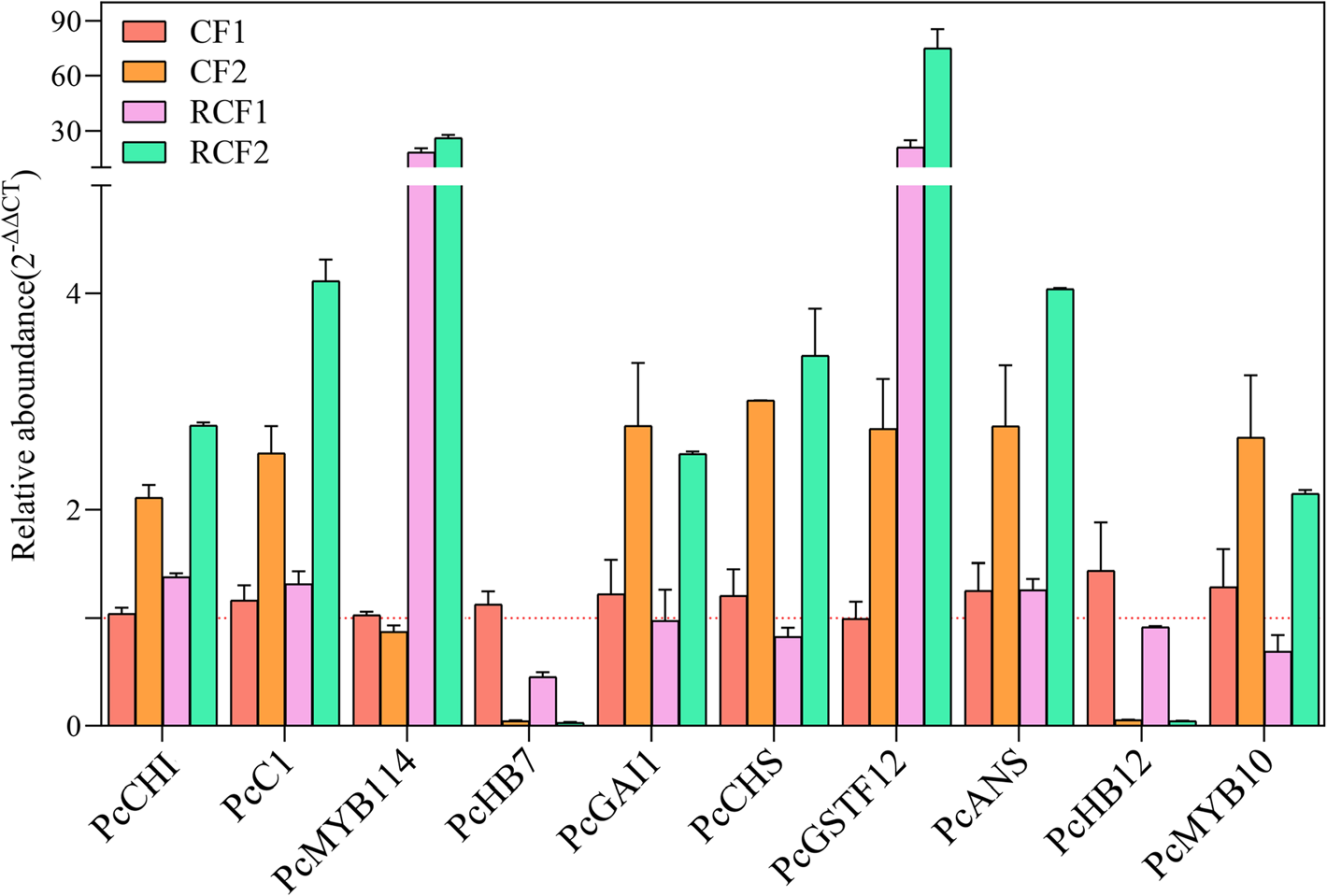
Transcript levels of anthocyanin- and procyanidin-related genes. Each experiment had three biological replicates

To validate the RNA-seq data, we conducted qPCR analyses of 10 of the anthocyanin and/or PA candidate genes: *PcCHI*, *PcC1*, *PcMYB114*, *PcHB7*, *PcGAI1*, *PcCHS*, *PcGSTF12*, *PcANS*, *PcHB12,* and *PcMYB10* (for gene IDs and primers, see Supplementary Table S9). The transcript profiles of all selected genes were very similar to those detected from the RNA-seq data (Fig. 4). The results showed that *PcGSTF12* was most up-regulated in comparison groups ‘RCF1 vs. CF1’ and ‘RCF2 vs. CF2’. This result was highly consistent with the results of RNA-seq, and provided further evidence for the crucial role of *PcGSTF12* in anthocyanin and PA accumulation in pear. Thus, we conducted further analyses to confirm the function of *PcGSTF12*.

### *PcGSTF12*-mediated anthocyanin and PA accumulation in pear

Our combined metabolite and transcriptomic analyses revealed a core set of genes closely correlated with pear anthocyanins and PAs, which strongly suggested that they play key roles in anthocyanin and PA accumulation in pear. To test this, we focused on the most up-regulated gene among the core set of anthocyanin and PA candidate genes, *PcGSTF12*, for functional analysis.

#### Functional analysis of *PcGSTF12*

**Fig. 5:**
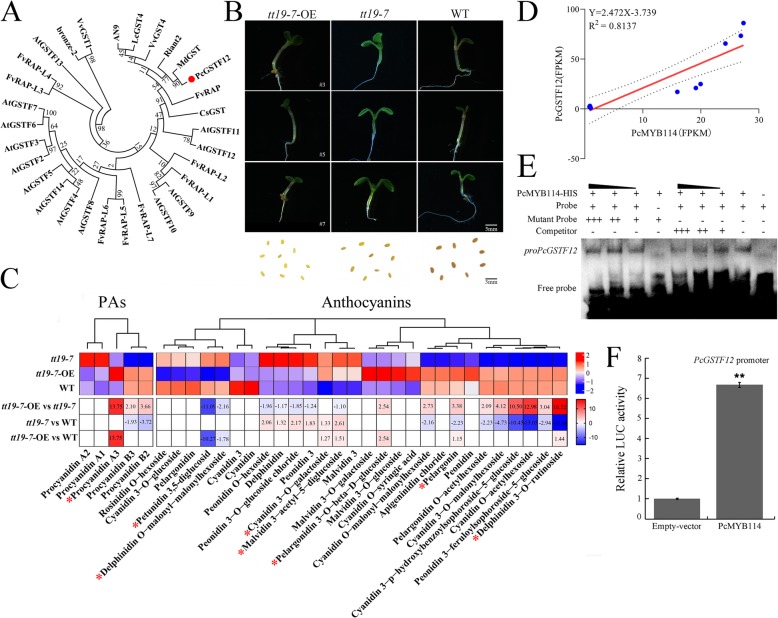
Functional analysis of *PcGSTF12* in anthocyanin and procyanidin (PA) accumulation. **a** Phylogenetic analyses of PcGSTF12 and its paralogs. The accession numbers are MdGST (AEN84869), Riant2 (KT312848), LcGST4 (KT946768), AN9 (Y07721), FvRAP-L1 (gene28763), VvGST4 (AAX81329), CsGST (ABA42223), AtGSTF2 (At4G02520), AtGSTF5 (At1G02940), AtGSTF3 (At2G02930), bronze-2 (AAV64226), FvRAP-L7 (gene10552), AtGSTF12/TT19 (At5G17220), AtGSTF14 (At1G49860), AtGSTF7 (At1G02920), VvGST1 (AAN85826), FvRAP (gene31672), FvRAP-L4 (gene10549), FvRAP-L2 (gene08595), FvRAP-L3 (gene22014), AtGSTF10 (At2G30870), FvRAP-L5 (gene10550), AtGSTF11 (At3G03190), AtGSTF13 (At3G62760), FvRAP-L6 (gene10551), AtGSTF8 (At2G47730), AtGSTF4 (At1G02950), AtGSTF6 (At1G02930), AtGSTF9 (At2G30860). **b** Phenotypes of *tt19–7*-OE seedlings and fresh seeds. **c** Heat map of anthocyanins and PAs of 7-day-old seedlings of *tt19–7* mutant, *tt19–7*-OE, and WT (wild-type). *tt19–7*-OE refers to 35S::*PcGSTF12* transgenic lines. Scale bars: 5 mm (B–C). Differentially accumulated metabolites between WT and *tt19–7*-OE marked by red star. **d** Correlation analyses between transcript levels of *PcGSTF12* and *PcMYB114*. **e** EMSA assays. Probe was biotin-labeled fragment containing MBS motif. Competitor probe was non-labeled probe. Mutant probe contained two nucleotide mutations. Competitors, and mutant probes at a 10×, 25×, and 50× molar excess were present (+) or absent (−) in each reaction. Black arrows indicate increasing multiples. **f** Effects of PcMYB114 on promoter activities as demonstrated by luciferase reporter assays. Empty-vector was used as a control. Values are means ± SD of three independent biological replicates. Statistical significance: **P < 0.01

The phylogenetic analysis showed that *PcGSTF12* is a homolog of *FvRAP* in strawberry, *Riant2* in peach, and *MdGST* in apple, all of which are in the phi subfamily [26] (Fig. 5a). Members of the phi subfamily are anthocyanin transporters. To test the potential role of *PcGSTF12* in anthocyanin accumulation, 35S:: *PcGSTF12* was transformed into the *Arabidopsis* mutant *tt19–7* (for primers, see Supplementary Table S9). The *tt19–7* plants showed a green hypocotyl phenotype, while the *tt19–7-*OE transgenic plants showed the red hypocotyl phenotype, like that of the wild type (WT) (Fig. 5b). However, the brown color of seed coats was not rescued in the *tt19–7*-OE lines (Fig. 5b). This result was consistent with the fresh seed phenotype obtained by transferring 35S:: *RAP-RFP* into *Arabidopsis tt19–7* [26].

To explore the role of *PcGSTF12* in anthocyanin and PA accumulation, we conducted a metabolite analysis using *Arabidopsis* seedlings. Three PAs and nine anthocyanins were significantly up-regulated, and three anthocyanins were significantly down-regulated in *tt19–7*-OE compared with *tt19–7*. Procyanidin A3, cyanidin O-acetylhexoside, delphinidin 3-O-rutinoside, and cyanidin 3-p-hydroxybenzoylsophoroside-5-glucoside were specifically up-regulated in *tt19–7*-OE compared with *tt19–7* (Fig. 5c). Five anthocyanins (malvidin 3-acetyl-5-diglucoside, pelargonidin 3-O-beta-D-glucoside, delphinidin 3-O-rutinoside, pelargonin, cyanidin 3-O-galactoside) and one PA (procyanidin A3) were significantly up-regulated, and two anthocyanins (petunidin 3, 5-diglucoside and delphinidin O-malonyl-malonylhexoside) were significantly down-regulated in *tt19–7*-OE compared with WT. Interestingly, a large amount of petunidin 3, 5-diglucoside was detected in WT but not in *tt19–7*-OE. In contrast, procyanidin A3 was detected only in *tt19–7*-OE. These results confirmed that *PcGSTF12* is responsible for anthocyanin and PA accumulation. Interestingly, its affinity for anthocyanins and PAs differed from that of *AtGSTs* in *Arabidopsis*. In particular, our results showed that *PcGSTF12* is responsible for the accumulation of procyanidin A3 but not petunidin 3, 5-diglucoside, opposite to the function of *AtGSTs* in *Arabidopsis*. *PcGSTF12* is a newly identified member of the phi GST family involved in anthocyanin and PA accumulation.

Next, we analyzed RNA-seq data to identify which genes were affected by *PcGSTF12* in the seedlings of *tt19–7*-OE vs. *tt19–7*. In total, we found 28 strongly affected genes, which encoded proteins involved in anthocyanin and PA biosynthesis, regulation, and transport (Supplementary Table S10). These results showed that *PcGSTF12* might not only be an anthocyanin and PA transporter, but may also participate in many other steps of anthocyanin and PA accumulation.

#### Upstream regulation of *PcGST12*

Correlation analyses showed that the transcript level of *PcGSTF12* was significantly correlated with that of *PcMYB114* (Fig. 5d). Further, several MYB-binding sites were found in the *PcGSTF12* promoter, indicating that *PcGSTF12* might be directly bound by, and regulated by, MYB transcription factors (Supplementary Table S11). Several R2R3-MYB genes are known to bind to MBS sites [27], and we found a MBS site within an 801-bp region upstream of the start codon. Thus, this MBS site was used in an EMSA assay (for primers, see Supplementary Table S9). The biotinylated probe was able to bind PcMYB114 protein, and the addition of a high concentration of cold probe significantly reduced the binding affinity of the biotinylated probe. To test whether *PcGSTF12* could be regulated by PcMYB114, we further carried out luciferase reporter assay (for primers, see Supplementary Table S9). The relative LUC activity of the *PcGSTF12* promoter was about 6-fold that of the empty vector control. These results showed that PcMYB114 could directly bind to the MBS site in the *PcGSTF12* promoter (Fig. 5e) and positively regulate its activity (Fig. 5f).

We also found many *cis*-acting elements involved in auxin-, ethylene-, and gibberellin-signaling in the *PcGSTF12* promoter. This indicated that *PcGSTF12* might be the common downstream target of R2R3-MYBs, and auxin, ethylene, and gibberellin signals that regulate the anthocyanin and PA pathways (Supplementary Table S11). Together, these results provide new clues about *PcGSTF12*-mediated anthocyanin and PA accumulation in pear.

## Discussion

We present the first genome-wide examination of anthocyanins, PAs, and the gene expression profiles of pear using young fruits of cv. ‘CF’ and its red mutant ‘RCF’. Through combined transcriptomic and metabolic analyses, we found a core set of 20 candidate genes related to anthocyanin and PA accumulation in pear. These findings increase our understanding of the molecular mechanism of anthocyanin and PA accumulation in pear, especially during the early stage of fruit development.

The core set of candidate genes for pear anthocyanin and PA accumulation includes six flavonoid structural genes: *PcCHI* (PCP027877, PCP021141, and PCP039844), *PcCHS* (PCP023048), *PcF3H* (PCP013732), and *PcANS* (PCP027029). CHI, CHS, and F3H are common to both the anthocyanin and PA biosynthetic pathways [6]. ANS can catalyze the conversion of (+)-catechin to cyanidin and a procyanidin [28]. Functional analyses have confirmed the roles of *PcCHI*, *PcCHS,* and *PcF3H* in anthocyanin accumulation [29]. The results of those studies and our study provide evidence that the patterns of anthocyanin and PA accumulation are conserved among different plants.

In plants, TFs play important roles in flavonoid regulation. The R2R3-MYBs make up one of the largest TF families [30], and most of them play important roles in flavonoid accumulation [31]. *PyMYB10* was the first R2R3-MYB TF identified to be involved in anthocyanin accumulation in pear [13]. Functional analyses have confirmed the roles of *PyMYB10.1* and *PyMYB114* in the regulation of anthocyanin accumulation in pear [14, 32], and the roles of *PbMYB10b* and *PbMYB9* in both anthocyanin and PA accumulation in pear [4]. In this study, the core set of candidate genes for anthocyanin and PA accumulation included four R2R3-MYB genes: *PcMYB3*, *PcMYB86*, *PcMYB111* and *PcMYB114*. Their transcript levels were strongly positively correlated with anthocyanins and PAs. Although *MYB114* was already known to function in anthocyanin accumulation in pear, it was unknown which particular metabolites were affected. Our results provide evidence that *PcMYB114* functions in the accumulation of six anthocyanin metabolites: pelargonidin 3-Glu, malvidin-3-galactoside chloride, cyanidin 3-O-malonylhexoside, oenin, delphinidin and peonidin O-hexoside (Supplementary Table S8). We also detected positive correlations between *PcMYB114* and procyanidin A, procyanidin B1, and procyanidin B3 (Supplementary Table S8), indicating that *PcMYB114* also functions in PA accumulation in pear. Except for *PcMYB114*, the other MYB genes *PcMYB3*, *PcMYB86,* and *PcMYB111* are newly identified as candidates involved in anthocyanin and PA accumulation in pear. Interestingly, these R2R3-MYBs were correlated with different anthocyanins and PAs, suggesting that they have undergone sub-specialization to play different and specific roles in anthocyanin and PA accumulation in pear.

Other TFs are also involved in anthocyanin and PA accumulation in pear. The HD family of TFs is unique to plants, and its members are proposed to play key roles in developmental processes such as root development, plant cell differentiation, fruit ripening, and leaf and flower senescence [33–35]. Members of the HD-Zip I and HD-Zip IV TF subfamilies also play key roles in anthocyanin accumulation. *ANTHOCYANINLESS2* (*ANL2*) was the first HD-Zip IV gene found to be involved in the tissue-specific accumulation of anthocyanins. In *Arabidopsis, ANL2* affects anthocyanin accumulation in subepidermal tissue on the abaxial side of rosette leaves and in epidermal tissue on the abaxial side of the leaves [36]. Recently, two HD-Zip I genes, *MdHB1* and *RhHB1* have been shown to influence anthocyanin accumulation in apple and rose, respectively. Overexpression of *MdHB1* led to reduced anthocyanin accumulation in apple flesh. MdHB1 was found to repress the transcription of *MdDFR* and *MdUFGT* indirectly by interacting with MdMYB10, MdbHLH3, and MdTTG1 [37]. Consistent with the results in apple, silencing of *RhHB1* in rose led to higher anthocyanin levels in the petals [38]. In this study, the core set of genes involved in anthocyanin and PA accumulation in pear included an HD TF gene, *PcKNAT1*. *KNAT1* is a member of the Class I KNOX HD gene family and is thought to play a role in meristem development and leaf morphogenesis [39]. We found that *PcKNAT1* was strongly positively correlated with five anthocyanins and seven PAs, implying that its function differs from the known functions of HD-Zip I and HD-Zip IV TFs in anthocyanin accumulation. Our results suggest that the Class I KNOX HD gene family might play important roles in anthocyanin and PA accumulation; this expands our knowledge of the function of the Class I KNOX HD gene family.

In our study, a bZIP TF gene, *PcbZIP1,* was positively and closely correlated with six anthocyanins and five PAs. The bZIP TFs harbor a highly conserved bZIP domain [40]. They are diverse transcriptional regulators, playing crucial roles in plant development, physiological processes, and biotic/abiotic stress responses [41]. Recently, two bZIP TFs, *MdHY5* and *MdbZIP44,* were shown to promote anthocyanin accumulation in apple [42, 43]. Our results provide further evidence that bZIP TFs act as positive regulators in anthocyanin accumulation. Thus, as well as functioning in anthocyanin accumulation, bZIP TFs may also play important roles in PA accumulation.

In this study, a WRKY TF gene, *WRKY28,* was closely negatively correlated with one anthocyanin and four PAs. *WRKY41–1* in *Brassica napus* and *WRKY75* in *Arabidopsis* are known repressors of anthocyanin biosynthesis [44, 45], while *MdWRKY40* in apple is a positive regulator of wounding-induced anthocyanin biosynthesis [46]. Our results are consistent with findings in *B. napus* and *Arabidopsis* and opposite to those in apple. It is possible that *WRKYs* have evolved different functions in anthocyanin accumulation, like the R2R3-MYB TFs. For example, some R2R3-MYB TFs are positive regulators of anthocyanin accumulation [13, 14], while others are negative regulators [19]. Further studies are required to elucidate the complex roles of *WRKYs* in PA accumulation in fruit trees and other plants.

Auxin is known to suppress anthocyanin accumulation, and has been shown to decrease the expression of anthocyanin regulatory and structural genes in apple and *Arabidopsis* [47, 48]. A recent study showed that the auxin factor MdARF13 negatively regulates the anthocyanin pathway in apple through interacting with MdMYB10 and binding to the promoter of *MdDFR*. The auxin/IAA protein MdIAA121 was shown to inhibit the recruitment of MdARF13 to the *MdDFR* promoter and weaken the inhibitory effect of MdAFR13 on anthocyanin accumulation [21]. Contrary to auxin, ethylene enhances anthocyanin and PA accumulation in pear and apple. In pear, the ethylene-responsive factor PyERF3 enhances anthocyanin accumulation via interacting with PyMYB114 [14]. In apple, MdERF1B regulates anthocyanins and PAs through interacting with MdMYB1, MdMYB9, and MdMYB11 [22]. In this study, an ethylene-responsive gene, *PcERF003*, was positively correlated with six anthocyanins and seven PAs, and an auxin-responsive gene, *PcIAA13*, was positively correlated with two anthocyanins and four PAs. These results are consistent with the previous findings that auxin represses anthocyanin accumulation via *IAA* genes, and ethylene enhances anthocyanin accumulation via *ERF* genes. Phytohormones play important roles in young fruit development. Young pear fruits contain high level of auxin, and low level of ethylene [49]. Further functional analyses of *PcERF003* and *PcIAA13* may help to elucidate the links between the effects of auxin and ethylene on anthocyanin and/or PA accumulation in young developing pear fruit.

Plant GSTs are encoded by a large gene family, and are soluble and abundant in the cytosol [26, 50, 51]. They can be divided into eight subgroups, among which the tau and phi classes play key roles in flavonoid transport [52]. *Bronze 2* (*bz2*) in maize was the first tau class GST reported to be involved in anthocyanin accumulation; *bz2* produces yellow skin kernels because of disabled anthocyanin transport into the vacuole [53]. Anthocyanin deposition is also affected by genes in the phi class, such as *AtGSTF12* in *Arabidopsis* [54, 55], *FvRAP* in strawberry [26], *CsGSTF1* in tea [52], and *VvGST4* in grapevine [56]. Interestingly, these phi-class GSTs show extensive functional diversification. For example, *AtGSTF12* plays a key role in both anthocyanin and PA accumulation in *Arabidopsis* [54], while *CsGSTF1* in tea functions only in anthocyanin accumulation [52]. Functional divergence of GSTs has arisen through nonsynonymous mutations, especially at key amino acid sites [51]. For example, a single amino acid mutation (Arg39 to Trp39) was found to be responsible for the high enzymatic activity of *Populus euphratica* PeGSTU30 [51]. Li et al. [57] showed that one Trp to Leu substitution at amino acid 205 in AtTT19 resulted in an anthocyanin-deficient phenotype in *Arabidopsis*. Recently, Luo et al. reported that a single nucleotide polymorphism (C to T) in the second exon of *FvRAP* dramatically decreased the anthocyanin level in the petiole and fruit of strawberry [26]. In our study, a phi-class GST gene, *PcGSTF12*, was among the core genes for anthocyanin and PA accumulation in pear. *PcGSTF12* was strongly associated with most of the studied metabolites in pear: seven anthocyanins and seven PAs. Similar to *AtGSTF12*, *PcGSTF12* was functionally characterized as an anthocyanin and PA carrier. Interestingly, we detected different affinities for anthocyanin and PA between *PcGSTF12* and *AtGSTs* in *Arabidopsis*. *PcGSTF12* plays an opposite role to that of *AtGSTs* in procyanidin A3 and petunidin 3, 5-diglucoside accumulation. These results suggest that phi-class *GSTs* have undergone extensive functional diversification during evolution. Furthermore, transformation with *PcGSTF12* affected genes encoding proteins involved in anthocyanin and PA biosynthesis, regulation, and transport. This functional analysis of *PcGSTF12* deepens our understanding of the roles of phi-class *GSTs* in anthocyanin and PA accumulation in pear and other plants.

We found that PcMYB114 can positively regulate *PcGSTF12* activity by directly binding to the MBS motif in its promoter. A recent previous study has shown that overexpression of *PyMYB114* in young pear fruit significantly enhance anthocyanin accumulation by upregulating anthocyanin structural genes *PyDFR*, *PyANS,* and *PyUFGT* [14]. However, whether *MYB114* functions in PA accumulation or affects anthocyanin and PA transportation are largely unknown. This result provides new evidence that *PcMYB114* functions both in anthocyanin and PA transportation in pear via regulating *PcGSTF12* activity, which may further affect anthocyanin and PA accumulation. Furthermore, we detected many *cis*-acting elements involved in auxin, ethylene, and gibberellin signaling in the promoter of *PcGSTF12*. Thus, we propose that *PcGSTF12* might be the common downstream target of auxin-, ethylene-, and gibberellin-mediated anthocyanin and PA accumulation pathways.

## Conclusions

In this study, we identified 4065 DEGs and 19 differentially expressed metabolites (12 anthocyanins and seven PAs) between young fruits of the green pear ‘CF’ and its red mutation ‘RCF’. Based on correlation analyses between DEGs and anthocyanins/PAs, we found 203 candidate genes for the accumulation of 10 anthocyanins and seven PAs. We further identified a ‘core set’ of 20 candidate genes for pear anthocyanin and PA accumulation. Of these, *PcGSTF12* was functionally characterized as an important anthocyanin and PA carrier in pear. We also identified an important pear anthocyanin and PA regulation node consisting of two core genes, *PcGSTF12* and *PcMYB114*. These results provide novel insights into pear anthocyanin and PA accumulation. The candidate genes for pear anthocyanin and PA accumulation presented here represent a valuable data set to guide future functional studies.

## Methods

### Plant materials

The cultivar ‘RCF’ is a typical red pear sport of cultivar ‘CF’ that was discovered in the USA. The fruit of ‘RCF’ is initially green, then changes quickly to red within 1 week after full bloom, and remains red until the fruit ripens. The coloration pattern of ‘RCF’ differs from that of most pear species, which color at the ripening stage. Thus, ‘CF’ and ‘RCF’ are ideal materials to study the molecular mechanism of anthocyanin and PA accumulation in young pear. The pear cultivars ‘CF’ and ‘RCF’ were cultivated in the experimental orchard of Yantai Academy of Agricultural Science, Shandong province, Yantai, China (37°5′N, 122°1′W). The fruits of ‘CF’ and ‘RCF’ were collected in 2017 from 6-year-old trees grafted onto *Pyrus betulaefolia* rootstocks. The different fruit skin color phenotypes of ‘CF’ and ‘RCF’ were visible at 5 DAFB. Thus, the regulation of anthocyanin and PA accumulation at this developmental stage of fruits is important for fruit coloration. We collected fruits of ‘CF’ and ‘RCF’ at two early developmental stages (2 DAFB and 5 DAFB) for further analyses. Briefly, fruits of ‘CF’ and ‘RCF’ with a similar green color were first sampled on 19 April 2017; these samples were collected at 2 DAFB, and were named ‘CF1’ and ‘RCF1’, respectively. Fruits with significant differences in color between ‘CF’ and ‘RCF’ were sampled on 22 April 2017; these samples collected at 5 DAFB were named ‘CF2’ and ‘RCF2’, respectively. In each experiment, skins from 100 fruits per replicate were collected. Three independent biological replicates were collected for analyses. The fruit skin samples were immediately frozen in liquid nitrogen and stored at − 80 °C until further metabolite, RNA-sequencing (RNA-Seq), and qPCR analyses.

### Metabolite extraction and separation

Metabolite extraction and separation were carried out as described by Wang et al. [23]. Briefly, the freeze-dried fruit skin was crushed into powder and then extracted overnight at 4 °C in 1.0 ml 70% aqueous methanol. Following centrifugation at 10, 000 g for 10 min, the extracts were filtered and analyzed by HPLC.

### Anthocyanin and PA identification and quantification

Anthocyanin and PA metabolites were annotated by comparisons against public databases including KNAPSAcK, MassBank, MoToDB, METLIN and HMDB, and were quantified using MRM as described by Wang et al. [23].

### Total RNA isolation and RNA-Seq analysis

Total RNA was isolated using Trizol reagent (Invitrogen, Carlsbad, CA, USA) and its integrity was evaluated using a 2100 Bioanalyzer (Agilent Technologies, Santa Clara, CA, USA). The mRNA was purified from high-quality total RNAs using oligo (dT) magnetic beads and then broken into short fragments with fragmentation buffer. The cDNA was synthesized using a cDNA Synthesis Kit (TaKaRa, Dalian, China) and linked to sequencing adapters at both ends. The cDNA libraries were sequenced using the Illumina sequencing system (HiSeq™ 2000, Illumina, San Diego, CA, USA). Clean reads were obtained using the NGS QC Toolkit [58]. Differential expression analysis was carried out using the DESeq R package (2012). The threshold for significant differential expression was *P* < 0.05, and |log 2 fold change| ≥ 1 was used to identify the differentially expressed genes (DEGs) between two different cDNA libraries. Gene Ontology (GO) and Kyoto Encyclopedia of Genes and Genomes (KEGG) enrichment analyses of DEGs were performed using the R platform as described elsewhere [59].

### Integrated metabolome and transcriptome analyses

Pearson’s correlation coefficients were calculated between the metabolome and transcriptome data. The coefficients were calculated from log2 (fold change) of each metabolite and log2 (fold change) of each transcript by the EXCEL program. Correlations with a coefficient of R^2^ > 0.8 were selected. Metabolome and transcriptome relationships were visualized using Cytoscape (version 2.8.2).

### qRT-PCR validation

The total RNA samples used for RNA-Seq were also used for cDNA synthesis using the PrimeScript™ RT Reagent Kit (TaKaRa) according to the manufacturer’s instructions. The qRT-PCR analyses were conducted as described previously [13] using the primers shown in Supplementary Table 1. *PcActin* was used as the reference gene. Three biological replicates were analyzed.

### Phylogenetic analysis

Phylogenetic tree analysis of PcGSTF12 and its homologs was carried out by MEGA 7 with bootstrap values calculated from 1000 replicate analyses.

### PcGSTF12 promoter analysis

The 2000-bp upstream sequence of *PcGSTF12* was analyzed using tools at the PlantCARE (http://bioinformatics.psb.ugent.be/webtools/plantcare/html/) and PLACE Signal Scan Search databases (https://www.dna.affrc.go.jp/PLACE/). The functional motifs are listed in Supplementary Table 10. A MYB-binding site (MBS) sequence CAACTG was found at − 801 bp in the promoter of *PcGSTF12* by PlantCARE. This MBS element is known to be a binding sequence of R2R3-MYBs [27, 60]. Therefore, we selected this MBS element sequence to further analyze the interaction between PcMYB114 and the promoter of *PcGSTF12* using electrophoretic mobility shift assays (EMSA) as described below.

### Electrophoretic mobility shift assay

The EMSA was performed using the LightShift Chemiluminescent EMSA Kit (Thermo Scientific, Waltham, MA, USA) as described by Jiang et al. [61]. The recombinant protein was purified using the Ni-agarose His-Tagged Protein Purification Kit (CWbiotech, Beijing, China).

### Luciferase reporter assay

Luciferase reporter assay was carried out as described by Wang et al. [62]. The CDS of *PcMYB114* was recombined into the pHBT-AvrRpm1 effector. The promoter of *PcGSTF12* was inserted into the pFRK1-LUC-nos reporter. The activities of LUC and GUS were detected using a Multimode Plate Reader (Victor X4, PerkinElmer, http://www.perkinelmer.com/).

### Ectopic expression of PcGSTF12 in *Arabidopsis*

For gene transformation, the CDS of *PcGSTF12* was recombined into the pRI101-AN vector and then transformed into *Agrobacterium tumefaciens* GV3101using the floral-dip method [63]. The T1 transgenic plants were selected on half-strength Murashige and Skoog (MS) solid medium with kanamycin. Kanamycin-resistant seedlings were grown in soil in a light incubator under a 16-h light/ 8-h dark photoperiod at 24 °C. Seven-day-old T2 seedlings were used for RNA-seq analysis and for anthocyanin and PA identification and quantification.

## Supplementary information

**Additional file 1:Table S1.** Comparison of anthocyanin and procyanidin composition between ‘CF’ and ‘RCF’. ‘CF1’ and ‘RCF1’ refer to fruits of ‘CF’ and ‘RCF’ collected at 2 days after full bloom (DAFB), respectively, ‘CF2’ and ‘RCF2’ refer to fruits of ‘CF’ and ‘RCF’ collected at 5 DAFB, respectively.

**Additional file 2:Table S2.** Overview of mapping of RNA-seq reads.

**Additional file 3:Table S3.** Differently expressed genes in four comparison groups: ‘RCF1’ vs. ‘CF1’, ‘RCF2’ vs. ‘CF2’, ‘RCF2’ vs. ‘RCF1’, and ‘CF2’ vs. ‘CF1’.

**Additional file 4:Table S4.** Gene Ontology (GO) functional annotations of DEGs in group 2–1 and group RCF-CF.

**Additional file 5:Table S5.** KEGG annotation of differentially expressed genes in group 2–1 and group RCF-CF.

**Additional file 6:Table S6.** Classification of 17 anthocyanin and procyanidin metabolites correlated with 203 DEGs.

**Additional file 7:Table S7.** Classification of 203 DEGs correlated with 17 anthocyanin and procyanidin metabolites.

**Additional file 8:Table S8.** Correlation analysis between 20 core genes and 10 anthocyanins and seven procyanidins.

**Additional file 9:Table S9.** List of primers used for qPCR analysis and making constructs.

**Additional file 10:Table S10.** RNA-seq analysis of candidate genes for anthocyanin and procyanidin accumulation in *tt19–7* and *tt19–7*-OE seedlings.

**Additional file 11:Table S11.***PcGSTF12* promoter analysis using PlantCARE (http://bioinformatics.psb.ugent.be/webtools/plantcare/html/) and PLACE Signal Scan Search database (https://www.dna.affrc.go.jp/PLACE/).

## Data Availability

Transcriptome sequencing data are available from the NCBI under the GEO accession number GSE146798. All data generated or analyzed during this study are included in this published article and its supplementary information files.

## Abbreviations

ANR: Anthocyanidin reductase
ANS: Anthocyanidin synthase
CF: Clapp Favorite
CHI: Chalcone isomerase
CHS: Chalcone synthase
DEGs: Differentially expressed genes
DFR: Dihydroflavonol reductase
F3H: Flavonoid 3-hydroxylase
GSTs: Glutathione S-transferases
LAR: Leucoanthocyanidin reductase
MATE: Multidrug and toxic compound extrusion protein
PAs: Procyanidins
RCF: Red Clapp Favorite
TFs: Transcription factors
UFGT: UDP-glucose flavonoid 3-Oglucosyl transferase)
WT: Wild-type

## References

[1] Tuan PA, Bai S, Yaegaki H, Tamura T, Hihara S, Moriguchi T (2015). The crucial role of *PpMYB10.1* in anthocyanin accumulation in peach and relationships between its allelic type and skin color phenotype. BMC Plant Biol.

[2] Xie M, Huang Y, Zhang Y, Wang X, Yang H, Yu O (2013). Transcriptome profiling of fruit development and maturation in Chinese white pear (*Pyrus bretschneideri* Rehd). BMC Genomics.

[3] Ngo T, Zhao Y (2009). Stabilization of anthocyanins on thermally processed red D’Anjou pears through complexation and polymerization. Food Sci Technol.

[4] Zhai R, Wang Z, Zhang S, Meng G, Song L, Wang Z (2016). Two MYB transcription factors regulate flavonoid biosynthesis in pear fruit (*Pyrus bretschneideri* Rehd.). J Exp Bot.

[5] Lesschaeve I, Noble AC (2005). Polyphenols: factors influencing their sensory properties and their effects on food and beverage preferences. Am J Clin Nutr.

[6] Koes R, Verweij W, Quattrocchio F (2005). Flavonoids: a colorful model for the regulation and evolution of biochemical pathways. Trends Plant Sci.

[7] Tanner GJ, Francki KT, Abrahams S, Watson JM, Larkin PJ, Ashton AR (2003). Proanthocyanidin biosynthesis in plants: purification of legume leucoanthocyanidin reductase and molecular cloning of its cDNA. J Biol Chem.

[8] Xie DY, Sharma SB, Paiva NL, Ferreira D, Dixon RA (2003). Role of anthocyanidin reductase, encoded by BANYULS in plant flavonoid biosynthesis. Science..

[9] Lam KC, Ibrahim RK, Behdad B, Dayanandan S (2007). Structure, function, and evolution of plant *O*-methyltransferases. Genome..

[10] Yin Y, Chen H, Hahn MG, Mohnen D, Xu Y (2010). Evolution and function of the plant cell wall synthesis-related glycosyltransferase family 8. Plant Physiol.

[11] Zhao J (2015). Flavonoid transport mechanisms: how to go, and with whom. Trends Plant Sci.

[12] Takos AM, Jaffé FW, Jacob SR, Bogs J, Robinson SP, Walker AR (2006). Light-induced expression of a MYB gene regulates anthocyanin biosynthesis in red apples. Plant Physiol.

[13] Feng S, Wang Y, Yang S, Xu Y, Chen X (2010). Anthocyanin biosynthesis in pears is regulated by a R2R3-MYB transcription factor PyMYB10. Planta..

[14] Yao G, Ming M, Allan AC, Gu C, Li L, Wu X (2017). Map-based cloning of the pear gene *MYB114* identifies an interaction with other transcription factors to coordinately regulate fruit anthocyanin biosynthesis. Plant J.

[15] Walker AR, Lee E, Bogs J, McDavid DA, Thomas MR, Robinson SP (2007). White grapes arose through the mutation of two similar and adjacent regulatory genes. Plant J.

[16] Deluc L, Barrieu F, Marchive C, Lauvergeat V, Decendit A, Richard T (2006). Characterization of a grapevine R2R3-MYB transcription factor that regulates the phenylpropanoid pathway. Plant Physiol.

[17] Deluc L, Bogs J, Walker AR, Ferrier T, Decendit A, Merillon JM (2008). The transcription factor VvMYB5b contributes to the regulation of anthocyanin and proanthocyanidin biosynthesis in developing grape berries. Plant Physiol.

[18] An XH, Tian Y, Chen KQ, Liu XJ, Liu DD, Xie XB (2015). MdMYB9 and MdMYB11 are involved in the regulation of the JA-induced biosynthesis of anthocyanin and proanthocyanidin in apples. Plant Cell Physiol.

[19] Zhou H, Lin-Wang K, Wang F, Espley RV, Ren F, Zhao J (2019). Activator-type R2R3-MYB genes induce a repressor-type R2R3-MYB gene to balance anthocyanin and proanthocyanidin accumulation. New Phytol.

[20] Ravaglia D, Espley RV, Henry-Kirk RA, Andreotti C, Ziosi V, Hellens RP (2013). Transcriptional regulation of flavonoid biosynthesis in nectarine (*Prunus persica*) by a set of R2R3 MYB transcription factors. BMC Plant Biol.

[21] Wang YC, Wang N, Xu HF, Jiang SH, Fang HC, Su MY (2018). Auxin regulates anthocyanin biosynthesis through the aux/IAA–ARF signaling pathway in apple. Hortic Res..

[22] Zhang J, Xu H, Wang N, Jiang S, Fang H, Zhang Z (2018). The ethylene response factor MdERF1B regulates anthocyanin and proanthocyanidin biosynthesis in apple. Plant Mol Biol.

[23] Wang Z, Cui Y, Vainstein A, Chen S, Ma H (2017). Regulation of fig (*Ficus carica* L.) fruit color: metabolomic and transcriptomic analyses of the flavonoid biosynthetic pathway. Front. Plant Sci.

[24] Cho K, Cho KS, Sohn HB, Ha IJ, Hong SY, Lee H (2016). Network analysis of the metabolome and transcriptome reveals novel regulation of potato pigmentation. J Exp Bot.

[25] Li Y, Fang J, Qi X, Lin M, Zhong Y, Sun L, et al. Combined analysis of the fruit metabolome and transcriptome reveals candidate genes involved in flavonoid biosynthesis in *Actinidia arguta*. Int J Mol Sci. 2018;19(5):1471. 10.3390/ijms19051471 PMC5983832 29762529

[26] Luo H, Dai C, Li Y, Feng J, Liu Z, Kang C (2018). Reduced anthocyanins in petioles codes for a GST anthocyanin transporter that is essential for the foliage and fruit coloration in strawberry. J Exp Bot.

[27] Chang C, Yu D, Jiao J, Jing S, Schulze-Lefert P, Shen QH (2013). Barley MLA immune receptors directly interfere with antagonistically acting transcription factors to initiate disease resistance signaling. Plant Cell.

[28] Wellmann F, Griesser M, Schwab W, Martens S, Eisenreich W, Matern U (2006). Anthocyanidin synthase from *Gerbera hybrida* catalyzes the conversion of (+)-catechin to cyanidin and a novel procyanidin. FEBS Lett.

[29] Wang Z, Meng D, Wang A, Li T, Jiang S, Cong P (2013). The methylation of the PcMYB10 promoter is associated with green- skinned sport in max red Bartlett pear. Plant Physiol.

[30] Riechmann JL, Heard J, Martin G, Reuber L, Jiang C, Keddie J (2000). *Arabidopsis* transcription factors: genome-wide comparative analysis among eukaryotes. Science..

[31] Dubos C, Stracke R, Grotewold E, Weisshaar B, Martin C, Lepiniec L (2010). MYB transcription factors in *Arabidopsis*. Trends Plant Sci.

[32] Feng S, Sun S, Chen X, Wu S, Wang D, Chen X (2015). PyMYB10 and PyMYB10.1 interact with bHLH to enhance anthocyanin accumulation in pears. PLoS One.

[33] Javelle M, Vernoud V, Rogowsky PM, Ingram GC (2011). Epidermis: the formation and functions of a fundamental plant tissue. New Phytol.

[34] Miao ZQ, Zhao PX, Mao JL, Yu LH, Yuan Y, Tang H (2018). HOMEOBOX PROTEIN52 mediates the crosstalk between ethylene and auxin signaling during primary root elongation by modulating auxin transport-related gene expression. Plant Cell.

[35] Manavella PA, Arce AL, Dezar CA, Bitton F, Renou JP, Crespi M (2006). Cross-talk between ethylene and drought signaling pathways is mediated by the sunflower Hahb-4 transcription factor. Plant J.

[36] Kubo H, Peeters AJM, Aarts MGM, Pereira A, Koornneef M (1999). ANTHOCYANINLESS2, a homeobox gene affecting anthocyanin distribution and root development in *Arabidopsis*. Plant Cell.

[37] Jiang Y, Liu C, Yan D, Wen X, Liu Y, Wang H (2017). MdHB1 down-regulation activates anthocyanin biosynthesis in the white-fleshed apple cultivar ‘granny smith’. J Exp Bot.

[38] Lü P, Zhang C, Liu J, Liu X, Jiang G, Jiang X (2014). RhHB1 mediates the antagonism of gibberellins to ABA and ethylene during rose (*Rosa hybrida*) petal senescence. Plant J.

[39] Lincoln C, Long J, Yamaguchi J, Serikawa K, Hake S (1994). A knotted1-like homeobox gene in *Arabidopsis* is expressed in the vegetative meristem and dramatically alters leaf morphology when overexpressed in transgenic plants. Plant Cell.

[40] Hurst HC (1995). Transcription factors 1: bZIP proteins. Protein Profile.

[41] Wang Z, Cheng K, Wan L, Yan L, Jiang H, Liu S (2015). Genome-wide analysis of the basic leucine zipper (bZIP) transcription factor gene family in six legume genomes. BMC Genomics.

[42] An JP, Qu FJ, Yao JF, Wang XN, You CX, Wang XF (2017). The bZIP transcription factor MdHY5 regulates anthocyanin accumulation and nitrate assimilation in apple. Hortic Res..

[43] An JP, Yao JF, Xu RR, You CX, Wang XF, Hao YJ (2018). Apple bZIP transcription factor MdbZIP44 regulates abscisic acid promoted anthocyanin accumulation. Plant Cell Environ.

[44] Devaiah BN, Karthikeyan AS, Raghothama KG (2007). WRKY75 transcription factor is a modulator of phosphate acquisition and root development in *Arabidopsis*. Plant Physiol.

[45] Duan S, Wang J, Gao C, Jin C, Li D, Peng D (2018). Functional characterization of a heterologously expressed *Brassica napus* WRKY41-1 transcription factor in regulating anthocyanin biosynthesis in *Arabidopsis thaliana*. Plant Sci.

[46] An JP, Zhang XW, You CX, Bi SQ, Wang XF, Hao YJ (2019). MdWRKY40 promotes wounding-induced anthocyanin biosynthesis in association with MdMYB1 and undergoes MdBT2-mediated degradation. New Phytol.

[47] Ji XH, Wang YT, Zhang R, Wu SJ, An MM, Li M (2015). Effect of auxin, cytokinin and nitrogen on anthocyanin biosynthesis in callus cultures of red-fleshed apple (*Malus sieversii f*. *niedzwetzkyana*). Plant Cell Tiss Org.

[48] Liu Z, Shi MZ, Xie DY (2014). Regulation of anthocyanin biosynthesis in *Arabidopsis thaliana* red *pap1-D* cells metabolically programmed by auxins. Planta..

[49] Cong L, Yue R, Wang H, Liu J, Zhai R, Yang J, et al. 2, 4-D-induced parthenocarpy in pear is mediated by enhancement of GA4 biosynthesis. Physiol Planta. 2018. 10.1111/ppl.12835. 10.1111/ppl.12835 30203555

[50] Dixon DP, Hawkins T, Hussey PJ, Edwards R (2009). Enzyme activities and subcellular localization of members of the *Arabidopsis* glutathione transferase superfamily. J Exp Bot.

[51] Yang Q, Han XM, Gu JK, Liu YJ, Yang MJ, Zeng QY (2019). Functional and structural profiles of GST gene family from three Populus species reveal the sequence-function decoupling of orthologous genes. New Phytol.

[52] Wei K, Wang L, Zhang Y, Ruan L, Li H, Wu L (2018). A coupled role for *CsMYB75* and *CsGSTF1* in anthocyanin hyperaccumulation in purple tea. Plant J.

[53] Marrs KA, Alfenito MR, Lloyd AM, Walbot V (1995). A glutathione S-transferase involved in vacuolar transfer encoded by the maize gene Bronze-2. Nature..

[54] Dixon DP, Skipsey M, Edwards R (2010). Roles for glutathione transferases in plant secondary metabolism. Phytochemistry..

[55] Sun Y, Li H, Huang JR (2012). *Arabidopsis* TT19 functions as a carrier to transport anthocyanin from the cytosol to tonoplasts. Mol Plant.

[56] Gomez C, Conejero G, Torregrosa L, Cheynier V, Terrier N, Ageorges A (2011). In vivo grapevine anthocyanin transport involves vesicle-mediated trafficking and the contribution of anthoMATE transporters and GST. Plant J.

[57] Li X, Gao P, Cui D, Wu L, Parkin I, Saberianfar R (2011). The *Arabidopsis tt19-4* mutant differentially accumulates proanthocyanidin and anthocyanin through a 3′ amino acid substitution in glutathione S-transferase. Plant Cell Environ.

[58] Patel RK, Jain M (2012). NGS QC toolkit: a toolkit for quality control of next generation sequencing data. PLoS One.

[59] Feng S, Sun J, Sun S, Wang Y, Tian C, Sun Q (2017). Transcriptional profiles underlying the effects of methyl jasmonate on apple ripening. J Plant Growth Regul.

[60] Mu RL, Cao YR, Liu YF, Lei G, Zou HF, Liao Y (2009). An R2R3-type transcription factor gene AtMYB59 regulates root growth and cell cycle progression in *Arabidopsis*. Cell Res.

[61] Jiang S, Chen M, He N, Chen X, Wang N, Sun Q (2019). *MdGSTF6*, activated by MdMYB1, plays an essential role in anthocyanin accumulation in apple. Hortic Res.

[62] Wang N, Xu H, Jiang S, Zhang Z, Lu N, Qiu H (2017). MYB12 and MYB22 play essential roles in proanthocyanidin and flavonol synthesis in red-fleshed apple (*Malus sieversii f. niedzwetzkyana*). Plant J.

[63] Clough SJ, Bent AF (1998). Floral dip: a simplified method for *Agrobacterium*-mediated transformation of *Arabidopsis thaliana*. Plant J.

