# Supplementary material for: Transcriptomic and metabolomic analysis provides insights into anthocyanin and procyanidin accumulation in pear
Source: BMC Plant Biol. 2020 Mar 27;20:129. doi: 10.1186/s12870-020-02344-0 (PMC7099803; doi:10.1186/s12870-020-02344-0)
Supplement: Supplementary file 2 — Additional file 2:Table S2. Overview of mapping of RNA-seq reads. [file 12870_2020_2344_MOESM2_ESM.doc]

| **Sample** | **Raw reads** | **Clean reads** | **Clean bases**  **(G)** | **Q30(%)** | **Uniquely mapped**  **(%)** | **Total mapped (%)** | **Genes Known**（FPKM ≥ 10） |
| --- | --- | --- | --- | --- | --- | --- | --- |
| **CF2** | **74016119±1558775** | **65094603±1407150** | **9.8±0.2** | **94.29±0.09** | **90.08±0.88** | **93.78±0.17** | **11004±60** |
| CF2_1 | 75134262 | 66109818 | 9.9 | 94.3 | 89.07 | 93.67 | 10936 |
| CF2_2 | 74678562 | 65685664 | 9.9 | 94.37 | 90.64 | 93.98 | 11030 |
| CF2_3 | 72235534 | 63488326 | 9.5 | 94.2 | 90.53 | 93.69 | 11047 |
| **CF1** | **55345977±8116228** | **47845123±7093413** | **7.2±1.1** | **93.83±0.16** | **91.29±0.44** | **93.40±0.22** | **10740±253** |
| CF1_1 | 48773120 | 41932730 | 6.3 | 93.67 | 91.66 | 93.53 | 11078 |
| CF1_2 | 64417846 | 55710414 | 8.4 | 93.84 | 91.41 | 93.53 | 11004 |
| CF1_3 | 52846966 | 45892224 | 6.9 | 93.99 | 90.81 | 93.15 | 10137 |
| **RCF2** | **58479545±4856967** | **50331164±4536249** | **7.5±0.7** | **93.95±0.19** | **90.67±0.63** | **93.65±0.10** | **11047±110** |
| RCF2_1 | 56791868 | 49656748 | 7.4 | 94.12 | 90.49 | 93.6 | 11169 |
| RCF2_2 | 54691544 | 46169880 | 6.9 | 93.75 | 91.37 | 93.76 | 11017 |
| RCF2_3 | 63955224 | 55166864 | 8.3 | 93.99 | 90.16 | 93.59 | 10955 |
| **RCF1** | **57467929±3363372** | **49065478±4114798** | **7.4±0.6** | **93.93±0.26** | **90.84±0.13** | **93.32±0.15** | **11135±102** |
| RCF1_1 | 55644548 | 47158560 | 7.1 | 93.89 | 90.87 | 93.15 | 11183 |
| RCF1_2 | 61349254 | 53787798 | 8.1 | 94.2 | 90.7 | 93.42 | 11018 |
| RCF1_3 | 55409984 | 46250076 | 6.9 | 93.69 | 90.95 | 93.39 | 11203 |
| Total | 735928712 | 637009102 | 95.6 | - | - | - | 14514 |
| Mean | 61327393 | 53084092 | 8.0 | 94.00 | 90.72 | 93.54 | 10981 |
| SD | 8871666 | 8344255 | 1.3 | 0.24 | 0.67 | 0.24 | 280 |

**Table S2.** Overview of mapping of RNA-seq reads.
